# Vitamin D Enhances Efficacy of Oral Nifedipine in Treating Preeclampsia with Severe Features: A Double Blinded, Placebo-Controlled and Randomized Clinical Trial

**DOI:** 10.3389/fphar.2017.00865

**Published:** 2017-11-24

**Authors:** Dan-Dan Shi, Yong Wang, Jun-Jun Guo, Ling Zhou, Na Wang

**Affiliations:** Cangzhou Central Hospital, Cangzhou, China

**Keywords:** preeclampsia, vitamin D, nifedipine, hypertension, pregnancy

## Abstract

Vitamin D (VD) has exhibited immunomodulatory role in the pathogenesis of preeclampsia. We hypothesize VD potentiate nifedipine treatment for preeclampsia by shortened the time to control blood pressure and prolong time before subsequent hypertensive crisis. We conduct a randomized trial of 683 primigravid women with preeclampsia, who were assigned to different treatment groups, either nifedipine+placebo or nifedipine+VD orally, by random after screening. Primary endpoints include time to control hypertension and time before another hypertensive crisis. Maternal adverse effects including nausea, vomiting, chest pain, mild headache, dizziness, maternal tachycardia, hypotension or shortness of breath, and neonatal parameters including birth weight and Apgar scores, as well as the minimum number of dosages needed to control hypertension were defined as secondary endpoints. Serum levels of cytokines tumor necrosis factor-α (TNF-α) and interleukin-10 (IL-10) were also examined. There was a marked reduction of the time required to control hypertension and a significant lengthening (*p* = 0.013) of the time before a new hypertensive crisis in participants received nifedipine+VD treatments (41.8 ± 18.3 min), in comparison with the nifedipine+placebo controls (61.1 ± 15.9 min). In women treated with nifedipine+VD, the minimum number of dosages needed to control hypertension was also lower. With regard to adverse effects, no statistical difference was observed between the two treatment groups. Moreover, treatment with VD increased IL-10 and reduced TNF-α serum levels. VD possesses the potential of serving as a safe and effective adjuvant to oral nifedipine in treating women with preeclampsia against hypertension, possibly through the upregulation of IL-10 and the downregulation of TNF-α.

## Introduction

Preeclampsia, which uniquely manifests during pregnancy after 20 weeks post-gestation, is a severe disorder affecting various systems (American College of Obstetricians and Gynecologists; Task Force on Hypertension in Pregnancy, 2013). As a leading cause of morbidity and mortality during pregnancy around the globe, preeclampsia induces severely elevated blood pressure and proteinuria. Therefore, it is essential to monitor and control the blood pressure of patients in the clinical treatment of preeclampsia (Magee et al., 2008; Abalos et al., 2014), in which anti-hypertensive drugs are widely used (Belfort et al., 1990).

Nifedipine, a blocker of Ca^2+^ channels (Fenakel et al., 1991), is commonly prescribed to preeclampsia patients as a first line anti-hypertensive drug (Fenakel et al., 1991; Magee et al., 2008; Duley et al., 2013). Nifedipine improves renal blood flow, thereby decreasing vascular resistance. It also yields increased urine output by suppressing anti-diuretic hormones. Hence, nifedipine has become a potent drug to effectively control maternal hypertension (Giannubilo et al., 2012). More importantly, it has been proven that nifedipine is safe with patients, in particular pregnant women (Clark et al., 2015). Treatment with nifedipine during the third trimester can effectively alleviate high blood pressure without any serious neonatal or maternal adverse effects (Childress and Katz, 1994).

Recent findings have implicated vitamin D (VD) in the pathogenesis of pregnancy-related hypertension and/or preeclampsia as a potential risk factor (Baker et al., 2010; Robinson et al., 2013; Adela et al., 2017). For example, VD deficiency increases risks of both mild and severe preeclampsia (Baca et al., 2016), causing the incidence of preeclampsia to increase as much as fivefold in Bangladesh population (Ullah et al., 2013). In women at high risk for preeclampsia, maternal VD level in the second trimester was found to be negatively correlated with risk of early-onset preeclampsia and preterm birth at <35 weeks (Gernand et al., 2016; Zhao et al., 2017). In line with these reports, therapies aiming at supplementing VD have yielded promising results in experimental animal hypertensive/preeclampsia models. In a rat hypertensive model without preeclampsia features, VD supplementation improved factors associated with preeclampsia, such as AT1-AA-induced downstream targets, and reduced blood pressure (Faulkner et al., 2017). In a rat model of preeclampsia, VD supplementation restores angiogenic balance and decreases tumor necrosis factor-α (TNF-α), and importantly, reduced blood pressure (Song et al., 2017). Taken together the abovementioned studies likely supported the potential of VD as an anti-hypertensive agent in the treatment of preeclampsia. However, to the best of our knowledge, the effect of VD hasn’t been investigated in a clinical setting.

Recently, the inflammatory process was implicated in the pathophysiology of preeclampsia (LaMarca et al., 2016). In addition, since development of preeclampsia was reported to be caused by defective trophoblastic invasion, probably through increased pro-inflammatory cytokine TNF-α and decreased IL-10 (Royle et al., 2009), and VD was also reported to act as an immune modulator by downregulating TNF-α and upregulating IL-10 (Mora et al., 2008; Akbar and Zacharek, 2011). Therefore in this current trial, we planned to supplement VD into oral nifedipine therapy among women with preeclampsia, and evaluate the treatment outcomes, especially time needed to lower blood pressure and time before another hypertensive crisis, as well as assess potential adverse effects.

## Materials and Methods

### Ethics Statement

This is a double-blinded, placebo-controlled, randomized clinical trial of women with preeclampsia. The present clinical trial was designed in conformity with the guidelines of Declaration of Helsinki. All experimental protocols were approved by the Ethical Committee of Cangzhou Central Hospital. All participants provided oral and written consent. This study was registered in Chinese Clinical Trial Registry Center (registration number: ChiCTR-IPR-17013313).

### Patient Screening

During January 2011 and December 2016, women with singleton pregnancy who were diagnosed of preeclampsia in Cangzhou Central Hospital participated in the present trial. Inclusion criteria were: (1) primigravid women; (2) Gestation age between 20 and 30 weeks (although expectant management of preeclampsia patients with severe features beyond 34 weeks is accepted in our hospital guideline, it may not be the standard of care in all institutions); (3) hypertensive disorders (≥150 mmHg systolic and/or ≥100 mmHg diastolic); (4) proteinuria (≥0.3 g protein/24 h). All patients were later excluded based on the following criteria: (1) history of heart failure during the course of the pregnancy; (2) history of smoking and/or diabetes, both of which could contribute to hypertension; (3) use of anti-hypertensive drugs and/or VD supplementation; (4) HELLP syndrome or chronic hypertension.

### Randomization Process and Drug Treatment

Eligible patients were divided into two groups using stratified permuted-block randomization method with diastolic blood pressure as a factor: (1) nifedipine+VD group (*n* = 298), given one capsule containing nifedipine (10 mg per capsule) and VD (200 IU per capsule) every 15 min orally, up to four doses, until blood pressure was equal to or below 150/100 mmHg; (2) nifedipine+placebo group (*n* = 304), given one capsule containing nifedipine (10 mg per capsule) plus glucose (20 mg per capsule) as placebo every 15 min orally, up to four doses, until blood pressure was equal to or below 150/100 mmHg. The therapy was discontinued once blood pressure reached the set level, or after four doses, whichever comes earlier. Capsules containing VD or placebo were prepared by researchers unaware of the group assignment, and appeared identical to mask the content to the patients.

### Definition of Endpoints

Primary endpoints included: (1) time needed to lower blood pressure of the patients to 150/100 mmHg; (2) time before another hypertensive crisis (blood pressure > 150/100 mmHg). Secondary endpoints included: (1) the number of dosages needed to lower blood pressure to 150/100 mmHg; (2) maternal adverse effects including nausea, vomiting, chest pain, mild headache, dizziness, maternal tachycardia, hypotension, or shortness of breath; (3) neonatal parameters including birth weight and Apgar scores; (4) parameters such as placenta weight, delivery modus, and incidences of intensive care unit visits of mother or newborn; (5) serum levels of TNF-α and IL-10. All measurements were carried out double-blindly.

### Anthropometrics

Body weight was measured on a digital scale with 0.1 kg accuracy and body height was measured on a stadiometer with accuracy of 0.1 cm while patients stood straight bare-footed in light clothing. Body mass index (BMI) was then calculated as body weight/(body height)^2^. Measurement of blood pressure was preformed using a digital blood pressure monitor with 0.1 mmHg accuracy.

### Adverse Effects

All patients were surveyed at the end of the trial on symptoms that might have occurred during the trial: nausea, vomiting, chest pain, mild headache, dizziness, maternal tachycardia, hypotension, or shortness of breath. Heart rates of the mother and the fetus were continually monitored throughout the therapy.

### Detection of Serum Levels of TNF-α and IL-10

10 mL of blood samples were collected from each patient after blood pressure was equal to or below 150/100 mmHg, or after four doses, whichever comes earlier. Whole blood was immediately centrifugated at 3,000 rpm for 10 min to separate the serum, which was stored at -70°C for further analysis. Serum levels of TNF-α and IL-10 were examined using ELISA kits specific for human TNF-α and IL-10 (Cusabio Biotech, Co., Ltd., China) according to the manufacturer’s protocol.

### Statistical Analysis

Sample size of treatment groups was determined using established statistical power analysis (Cohen, 1992). Differences between means of each compared treatment groups were divided by the standard deviation to determine the standardized effect size (>2.0), then using 5% as significance level 90% power, the minimum required sample size was less than numbers used in the study. All data were determined to be normally distributed by Kolmogorov–Smirnov test. Statistical differences between treatments were determined using Student’s *t*-test or Pearson chi-square test, whichever was appropriate. *p*-Values less than 0.05 were considered significant. All analysis was performed using SPSS 18.0 (SPSS, Inc., United States).

## Results

**FIGURE 1:**
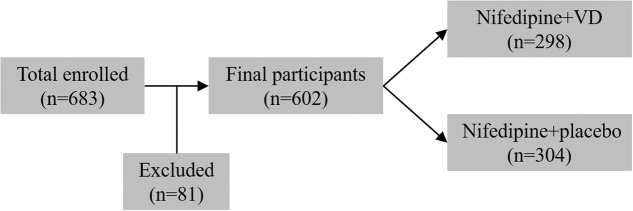
Study design flowchart.

From January 2011 to December 2016, 683 primigravid women (age 31–37) with preeclampsia and singleton pregnancy were enrolled in the current trial, among which 81 participants were later excluded. The remaining 602 eligible participants were assigned to two treatment groups in a random manner (**Figure 1**). 298 patients received nifedipine+VD orally, and 304 patients received oral nifedipine+placebo, a single dosage every 15 min until their blood pressure were equal to or lower than 150/100 mmHg. Anthropometrical parameters, such as maternal age, gestational age at term, heart rate, BMI, and systolic and diastolic blood pressures, are presented in **Table 1**. The difference between patients from two treatment groups was not statistically significant.

**Table 1:** Characteristics of patients from the two treatment groups.

	Nifedipine+placebo	Nifedipine+VD
Characteristics	(*n* = 304)	(*n* = 298)
Maternal age (year)	33.2 ± 6.7	32.7 ± 5.2
Gestation age at enrollment (week)	24.3 ± 4.1	25.6 ± 3.4
Gestation age at delivery (week)	36.4 ± 2.1	37.1 ± 2.9
Pre-treatment systolic blood pressure (mmHg)	169.0 ± 11.2	171.1 ± 13.4
Pre-treatment diastolic blood pressure (mmHg)	108.9 ± 12.7	110.2 ± 13.1
Pre-treatment heart rate (/min)	83.8 ± 8.2	84.5 ± 9.0
Post-treatment systolic blood pressure (mmHg)	159.6 ± 10.8	143.4 ± 7.6
Post-treatment diastolic blood pressure (mmHg)	101.9 ± 9.5	97.1 ± 9.2
Post-treatment heart rate (/min)	81.6 ± 9.1	80.8 ± 10.6
Body mass index (kg/m^2^)	24.1 ± 2.7	23.9 ± 3.2

*Data were shown as mean ± SD.*

**Table 2** summarized the primary endpoints of the two groups. In the group given nifedipine+VD the time required to control hypertension was 41.8 ± 18.3 min; whereas it was 61.1 ± 15.9 min in the group given nifedipine+placebo, markedly longer compared to the experimental group (*p* = 0.013). Further, group the time before another hypertensive crisis after blood pressure was controlled in the nifedipine+VD group was 8.1 ± 2.2 h, statistically longer than 4.8 ± 2.6 h in the nifedipine+placebo (*p* = 0.017).

**Table 2:** Efficacy of the two treatments in controlling blood pressure among women with preeclampsia.

Primary	Nifedipine+placebo	Nifedipine+VD	
endpoints	(*n* = 304)	(*n* = 298)	*p*-Value
Time to control blood pressure (min)	61.1 ± 15.9	41.8 ± 18.3	0.013
Time before a new hypertensive crisis (hour)	4.8 ± 2.6	8.1 ± 2.2	0.017

*Data were shown as mean ± SD. Criteria for stopping therapy was blood pressure ≤ 150/100 mmHg. Another hypertensive crisis was defined as blood pressure > 150/100 mmHg.*

**FIGURE 2:**
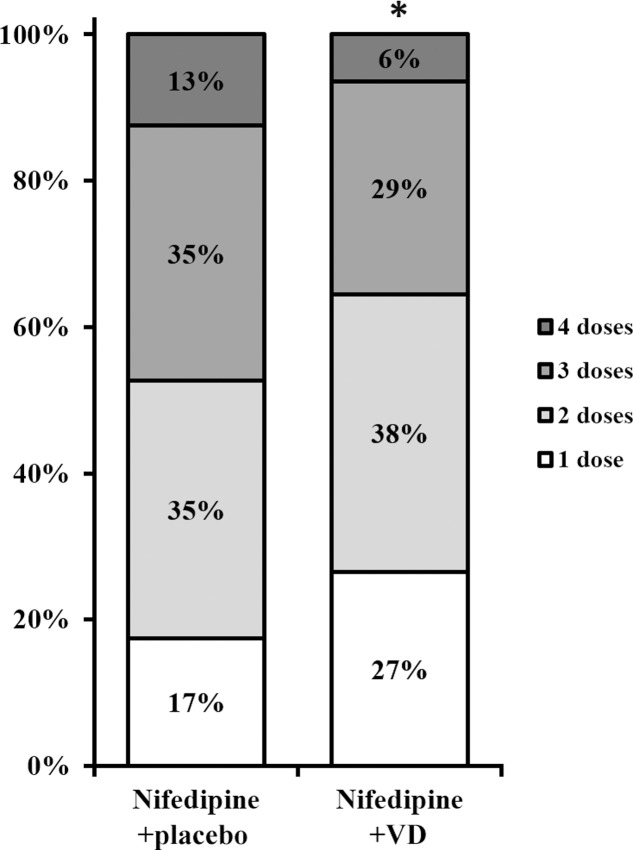
Number of doses needed to control blood pressure in two groups of patients. Percentages do not add up to 100 due to rounding. ^∗^All dose categories *p* < 0.05, nifedipine+VD compared to the nifedipine+placebo group.

Secondary endpoints following both treatments were also assessed. The number of dosages needed to effectively control blood pressure was greatly lower in patients who received nifedipine+VD compared to nifedipine+placebo group, as demonstrated in **Figure 2** (all dose categories *p* < 0.05). Further, both maternal and neonatal adverse effects, were evaluated at the end of the trial as summarized in **Table 3**. No statistical difference with regard to maternal adverse effects was observed between the two treatment groups, including vomiting, dizziness, nausea, mild headache, chest pain, maternal tachycardia, hypotension, or shortness of breath. Neither was there any difference in terms of neonatal parameters, such as birth weight and newborn Apgar scores. Moreover, delivery parameters, including placenta weight, mode of delivery, incidences of intensive care unit stay of mother or newborn were also analyzed (**Table 4**) and yielded no significant difference between the two treatments. No neonatal death was observed in the study.

**Table 3:** Maternal adverse effects of the two treatments.

Secondary	Nifedipine+placebo	Nifedipine+VD	
endpoints	(*n* = 304)	(*n* = 298)	
No adverse effect	262 (86.2%)	259 (86.9%)	*NS*
Nausea	13 (4.3%)	11 (3.7%)	*NS*
Vomiting	7 (2.3%)	8 (2.7%)	*NS*
Maternal tachycardia	7 (2.3%)	5 (1.7%)	*NS*
Mild headache	5 (1.6%)	5 (1.7%)	*NS*
Dizziness	4 (1.3%)	5 (1.7%)	*NS*
Chest pain	2 (0.7%)	3 (1.0%)	*NS*
Hypotension	2 (0.7%)	1 (0.3%)	*NS*
Shortness of breath	2 (0.7%)	1 (0.3%)	*NS*

*Percentages do not add up to 100 due to rounding. *NS*, not significant.*

**Table 4:** Delivery parameters and neonatal complications of the two treatments.

Delivery	Nifedipine+placebo	Nifedipine+VD	
parameters	(*n* = 304)	(*n* = 298)	
Placenta weight (g)	481.6 ± 91.3	492.3 ± 97.6	*NS*
**Mode of delivery**			
*Spontaneous vaginal delivery*	141 (46.4%)	133 (44.6%)	*NS*
*Vacuum extraction*	68 (22.4%)	64 (21.5%)	*NS*
*Cesarean section*	95 (31.3%)	101 (33.9%)	*NS*
**Intensive care unit stay**			
*Mother*	9 (3.0%)	6 (2.0%)	*NS*
*Newborn*	5 (1.6%)	5 (1.7%)	*NS*
**Delivery outcomes**			
Birth weight (kg)	3.26 ± 0.51	3.11 ± 0.37	*NS*
**Apgar scores**			
>6	241 (79.3%)	240 (80.5%)	*NS*
4–6	63 (20.7%)	58 (19.5%)	*NS*

*Percentages do not add up to 100 due to rounding. *NS*, not significant.*

**FIGURE 3:**
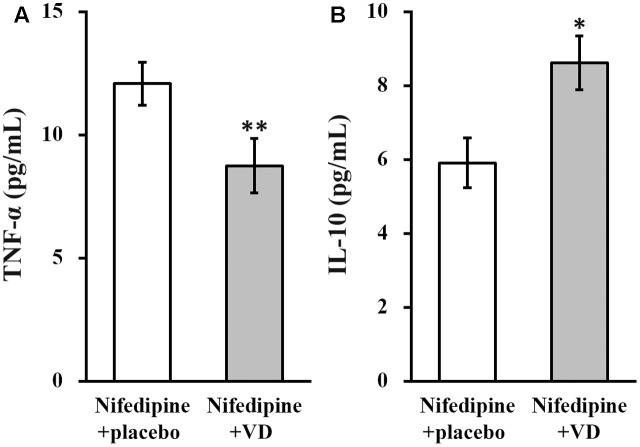
Serum levels (pg/mL) of TNF-α **(A)** and IL-10 **(B)** in the two groups of patients. Data were shown as mean ± SD (pg/mL). ^∗∗^*p* < 0.01, ^∗^*p* < 0.05, nifedipine+VD compared to the nifedipine+placebo group.

Serum levels of TNF-α and IL-10 were examined in all patients from both the treatment groups. As shown in **Figure 3**, in nifedipine+VD group, serum level of TNF-α was significantly reduced (**Figure 3A**), whereas that of IL-10 was significantly elevated (**Figure 3B**).

## Discussion

Based on the reported function of VD against hypertension, we included VD in our clinical trial among women with preeclampsia, to examine whether VD could synergize with oral nifedipine to alleviate hypertension in preeclampsia. Our findings demonstrated that use of oral nifedipine and VD combined greatly improved the primary as well as the secondary endpoints, compared to nifedipine and placebo. Specifically, in the nifedipine+VD group, the time to control hypertension was reduced, time before another hypertensive crisis was delayed, and number of dosages needed to control hypertension was lower. Furthermore, we did not observe any serious adverse effects, either maternal or neonatal, following conjunctional use of VD, demonstrating the clinical safety of VD in pregnant patients.

Controlling severe range blood pressure in the management of preeclampsia is essential for reducing both maternal and fetal complications (Rey et al., 1997; Brown et al., 2000; Shi et al., 2015). Currently, nifedipine is commonly used for hypertensive management in patients with preeclampsia (Magee et al., 2008; Duley et al., 2013). Compared to other anti-hypertensive drugs, oral nifedipine is safer, less expensive and more effective in achieving comparable anti-hypertensive effects in preeclampsia (Fenakel et al., 1991; Aali and Nejad, 2002).

Despite of the fact that drugs for alleviating hypertension are widely available, patients with preeclampsia are often faced with the dilemma that effective anti-hypertensive compounds may not be safe for pregnant individuals, particularly for oral medications. Therefore, an oral agent with proved safety and efficacy to assist oral nifedipine therapy will definitely benefit the treatment of severe preeclampsia. VD is a commonly taken as dietary supplement, and reportedly helpful in a number of pregnancy-related complications such as gestational diabetes mellitus (Li and Xing, 2016).

Moreover, the mechanism accountable for the therapeutic actions of VD in preeclampsia remains largely unclear. A recent study has proposed that, in preeclampsia affected pregnancy, deficiencies in VD and long chain polyunsaturated fatty acid metabolism might disturb the one carbon cycle, leading to altered feto-placental growth and development in preeclampsia (Nandi et al., 2017). On the other hand. It was suggested by Reslan colleagues that systemic effects of preeclampsia may be linked to reduced calcium absorption in VD-deficient individuals (Reslan and Khalil, 2010). In addition, since development of preeclampsia was reported to be caused by defective trophoblastic invasion, probably through increased pro-inflammatory cytokine TNF-α and decreased anti-inflammatory IL-10 (Royle et al., 2009). In this context, VD was also reported to act as an immune modulator by downregulating TNF-α and upregulating IL-10 (Mora et al., 2008; Akbar and Zacharek, 2011). Consistent with previous studies, we also found that in patients from the nifedipine+VD group, serum level of TNF-α was significantly downregulated, whereas serum level of IL-10 was greatly upregulated, compared with patients from the nifedipine+placebo group. In this context, the previously established rat model of preeclampsia (Song et al., 2017) could serve as a useful platform to further investigate the mechanism explaining the shorter duration and fewer doses of the combinational therapy to achieve blood pressure control observed in human patients in our current study.

Nevertheless, this study has some limitations. First, the data of adverse maternal effects was based on patient recall rather than objective measures, which limited the significance and accuracy of this data. Second, dose effect of VD on hypertension and cytokine regulation was not adequately investigated in the current study, which also limited the understanding of VD action on the inflammatory process. Third, the expectant management of preeclampsia patients with severe features beyond 34 weeks is may not be the standard of care in all institutions, which limited the generalizability of the data. Last, the study population was entirely comprised of primips without preexisting hypertension or diabetes mellitus, who were treated at an atypical blood pressure threshold of severe hypertension, which poses another potential issue in the generalizability of the study.

In summary, our data from the current randomized, double blinded and placebo-controlled clinical trial is the first report on the potency and safety of VD serving as a potential adjuvant to oral nifedipine to enhance the efficacy of therapies against hypertension in women with preeclampsia. Importantly, this beneficial action of VD is likely mediated by downregulating TNF-α and upregulating IL-10. Future study is warranted to investigate the molecular mechanism of VD action on downregulating TNF-α and upregulating IL-10 during preeclampsia.

## Author Contributions

Did the experiments and analyzed the data: D-DS, YW, J-JG, and LZ. Wrote the manuscript and designed the study: NW.

## Conflict of Interest Statement

The authors declare that the research was conducted in the absence of any commercial or financial relationships that could be construed as a potential conflict of interest.
